# Squamous cell carcinoma of tongue in a renal transplant recipient

**DOI:** 10.4103/0971-5851.65336

**Published:** 2009

**Authors:** Pavan Malleshappa, Manjurhusen Aghariya, Chandralekha Tampi, Bharat V. Shah

**Affiliations:** *Department of Nephrology, Lilavati Hospital and Research Centre, Bandra West, Mumbai - 400 050, India*

**Keywords:** *Hemiglossectomy*, *modified neck dissection*, *renal transplant*, *squamous cell carcinoma*

## Abstract

The overall incidence of malignancies in post renal transplant recipients is reported to be approximately 5 to 6%. Malignancies are significant complications after transplantation. Common malignancies include malignancies of the skin and post-transplant lymphoproliferative disorder (PTLD). Squamous cell carcinoma of the tongue is very rare. We present a case of squamous cell carcinoma of the tongue developing nine years after renal transplantation, in a 30-year-old man. He underwent left hemiglossectomy initially and then modified neck dissection. His graft function continues to remain stable.

## INTRODUCTION

For renal transplant recipients, the risk of malignancy is considerably greater than in the general population.[1] In most cases, *de novo* cancer develops; in others, cancer is transferred with a donor organ, and occasionally there is recurrence of the pre-existing cancer in a recipient. Squamous cell carcinomas mostly of the skin, lip, cervix, and rarely lung, have been reported in renal transplant recipients. These make up the bulk of the epithelial malignancies. Occurrence of squamous cell carcinoma of the tongue in a renal transplant recipient is very rare. Our patient developed squamous cell carcinoma nine years after a well functioning renal transplant and had an excellent graft function throughout the course of his illness.

## CASE REPORT

**Figure 1:**
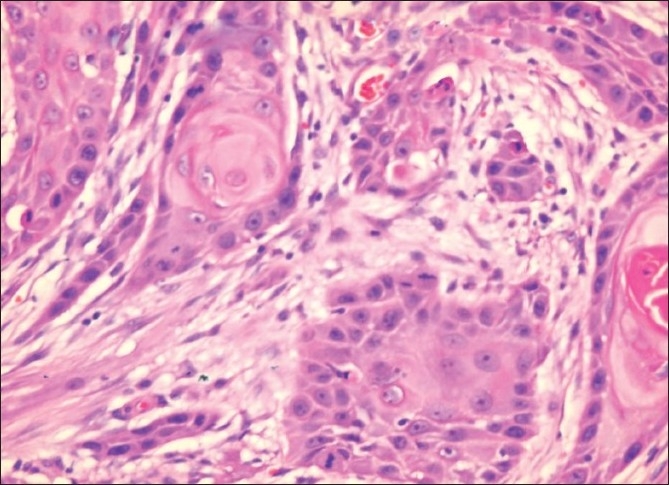
Well-differentiated squamous cell carcinoma of the tongue

A 30-year-old gentleman received a live related kidney transplant in 2000. His intra- and postoperative course was uncomplicated. He was treated with azathioprine, cyclosporine, and prednisolone. Good renal function was achieved within a week after transplantation. Nine years later, the patient, entirely well in the interim, presented with a small non-healing ulcer on the left midlateral margin of the tongue. He was taken for a biopsy of that non-healing ulcer. A frozen section revealed a well-differentiated squamous cell carcinoma, for which he underwent left hemiglossectomy. Histopathology revealed a well-differentiated squamous cell carcinoma; the maximum depth of the invasion was 1.9 cm, with tumor-free excisional margins [Figure 1]. Lymphatic and blood vessel invasion were not seen. The patient apparently never used to smoke or consume alcohol and denied any history of chewing tobacco. He had no history of wearing dentures, nor were there any identifiable factors, which may have contributed to chronic irritation.

He was kept under regular and frequent follow-ups. His immunosuppressive regimen was also changed from azathioprine, cyclosporine, and prednisolone to an everolimus and prednisolone combination, after left hemiglossectomy. His renal function continued to be stable. He noticed a painful, small swelling in his neck, just under his left mandible weeks after the surgery. Following this, an MRI of the neck was performed, which revealed enlarged left jugulodigastric lymph nodes, measuring 1.5 × 1.0 cm, without any evidence of local recurrence. He underwent left modified neck dissection, histopathology of which confirmed the presence of metastatic well-differentiated squamous cell carcinoma in the nodes. Polymerase chain reaction (PCR) for human papillomasvirus; (HPV) showed presence of HPV in the tumor tissue.

## DISCUSSION

Renal transplantation is an effective therapy for patients with otherwise terminal renal disease. However, it is not without certain attendant problems. One well-recognized complication is the increased risk of the development of malignant tumors in such patients. This may be largely related to the immunosuppressive therapy consistently given to avert graft rejection. Based on the comprehensive, long-term data from the Australian and New Zealand Dialysis and Transplantation Registry (ANZDATA Registry), 10 years after receiving a renal allograft, approximately 10% of all patients will develop cancer. After 20 years, this figure increases to approximately 25%, and after 30 years to approximately 40%.[2] The risk of cancer development is greater in patients who are older when they undergo transplantation. For men who are younger than 35 years at the time of the first kidney transplantation, the adjusted risk of developing cancer after 10 years is 4.2, whereas, for men who are 55 years old or older at the time of transplantation, it is 24.6.[2]

Skin malignancies are the most common types of cancer developing in renal transplant recipients and are a particular problem in parts of the world where predominantly white populations are exposed regularly to high intensity solar UV light.[3] In addition to this, many other malignancies occur in renal transplant recipients with increased frequency relative to the general population. These include Kaposi‘s sarcoma, hepatoma, and various malignancies of the gastrointestinal tract, particularly the esophagus and the large bowel. Very few cases of carcinoma of the tongue in a renal transplant recipient have been reported. In the 2004 ANZDATA report, the absolute cancer risk was determined for 14,354 patients who received a first renal transplant between 1963 and 2003. None of the recipients were reported to develop squamous cell carcinoma of the tongue.

Squamous cell carcinoma of the tongue is the most frequent intraoral malignancy.[4] It occurs more often in males and usually in the sixth to eighth decades, with only 11% occurring in the third and fourth decades. Smoking, chronic irritation, tobacco chewing, and HPV are believed to play an etiologic role. Numerous mechanisms are likely to contribute to the increased risk of cancer in immunosuppressed renal allograft recipients. The dominant factors are believed to be impaired immune surveillance for neoplastic cells, depressed antiviral immune activity, leading to viral re-activation, chronic antigenic stimulation, environmental factors, and direct neoplastic action of immunosuppressive drugs.

The development of carcinoma of the tongue in such a young patient, nine years after transplantation, in the absence of tobacco use or chronic irritation, suggests that the transplantation-immunosuppression experience may have reactivated the HPV virus and contributed to the development of carcinoma in this case.
